# Is Quantification of Measurable Clonal Plasma Cells in Stem Cell Grafts (gMRD) Clinically Meaningful?

**DOI:** 10.3389/fonc.2022.800711

**Published:** 2022-02-23

**Authors:** Guldane Cengiz Seval, Meral Beksac

**Affiliations:** Department of Hematology, School of Medicine, Ankara University, Ankara, Turkey

**Keywords:** multiple myeloma, autologous stem cell transplant (ASCT), minimal residual disease (MRD), graft contamination, clonal plasma cells

## Abstract

With the introduction of more effective novel therapies, the prognosis of multiple myeloma (MM) has improved significantly over the past decade, resulting with a significant proportion of patients achieving durable remissions that may reach even more than 10 years. Several studies demonstrated that the real prognostic value of complete remission (CR) relies on sustained undetectable minimal residual disease (MRD). Additionally, advances in MRD detection methods used for the detection of clonal plasma cells (cPC) inside or outside the bone marrow have also improved the value of MRD. The use of peripheral blood for MRD detection could be an effective method that overcomes the spatial heterogeneity and invasive intervention with recurrent bone marrow aspirations. During the last two decades, many groups have investigated the role of circulating plasma cells (CPCs) at diagnosis. As also presented by multiple groups during the recent ASH 2021 annual meeting, CPCs are becoming recognized as an independent prognostic factor. In addition, measurement of post-induction residual plasma cells in the stem cell graft is identified as another option for MRD assessment. Earlier studies in the era of less intensive induction regimens attempts to analyze the level of CPC contamination in the graft was shown to contribute to myeloma relapse and progression. According to these recent results, higher graft purity has been found to be in concordance with deeper responses. As expected, graft minimal residual disease (gMRD) may reflect the efficacy of induction as an additional response assessment tool. Although gMRD is a non-invasive approach, it has not gained sufficient support for routine use. In view of the hurdles related to monoclonal protein assessments, high-sensitivity cellular component measurement continues to possess its value as an end point for therapeutic efficacy. In this review, we will present a structural framework for MRD testing in peripheral blood stem cell autografts in MM and review the clinical integration into MM management.

## Introduction

The prognosis for patients with multiple myeloma (MM) has improved significantly over the past decade due to the use of more effective novel therapies and an increasing proportion of patients achieving durable remissions. Several studies demonstrated that the real prognostic value of complete remission (CR) relies on sustained undetectable minimal residual disease (MRD) (1–6). Additionally, MRD detection methods used for the detection of clonal plasma cells (cPCs) inside or outside the bone marrow have also improved. The use of peripheral blood for MRD detection could be an effective method that overcomes the spatial heterogeneity and invasive intervention with recurrent bone marrow aspirations.

Since cPCs may harbor sites outside of bone marrow or spread unevenly throughout the body, simultaneous measurement of disease activity within intramedullary and extramedullary compartments is required. Circulating plasma cells (CPCs) either at diagnosis or during mobilization can be detected in the peripheral blood among a significant number of myeloma patients (7–13). Although it can be simply a reflection of a tumor mass, it may also represent heterogeneity in the biology of the disease. Detection of CPCs in the blood may identify a status of independence from adherence to or dependence on the bone marrow microenvironment leading to a more aggressive disease. The prognostic significance of CPCs as an independent biomarker has been well established at the time of diagnosis (8, 14, 15). In addition, persistence of CPCs following treatment predicts shorter progression-free survival (PFS) and overall survival (OS) rates independent of patient age, cytogenetics, the type of therapy, or the depth of response achieved (16–19) (**Table 1**).

**Table 1:** Studies analyzing the impact of circulating PCs in the peripheral blood.

Reference	Study design	Number of patients	Detection method	Cutoff of cPCs	Time point assessment	Follow-up	Survival outcomes	HR	95% CI	p value
Witzig et al. (20)	Prospective	254	IFM	3 × 10^6^/L	At diagnosis	Not reported	OS	2.05	1.45–2.91	<0.01
Gertz et al. (21)	Prospective	33	IFM	0.2 × 10^6^/L	Pre-AHCT	Not reported	RFS OS	1.58 1.26	0.52–4.80 0.34–4.68	0.0057 0.0078
Nowakowski et al. (9)	Prospective	302	MFC	5 × 10 ^-3^	At diagnosis	33.5 months	OS	1.42	1.01–1.99	<0.001
Dingli et al. (16)	Prospective	246	MFC	5 × 10 ^-3^	Pre-AHCT	34 months	TTP OS	1.48 1.64	1.09–1.99 1.10–2.43	<0.001 0.005
Peceliunas et al. (22)	Prospective	42	MFC	2 × 10^-6^	At relapse	21 months	OS	2.33	1.01–5.36	
Korthals et al. (23)	Prospective	21	qPCR	Internal control	Pre-AHCT	45 months	EFS OS	1.53 1.76	0.18–13.36 0.12–25.79	0.6 0.5
Korthals et al. (23)	Prospective	32	qPCR	Internal control	Post-AHCT	45 months	EFS OS	4.41 5.88	1.56–12.48 1.03–33.59	0.004 0.03
Gonsalves et al. (10)	Retrospective	157	MFC	27 × 10^-4^	At diagnosis	Not reported	TTNT OS	1.85 3.16	1.07–3.16 1.43–7.08	<0.001 <0.001
Gonsalves et al. (24)	Retrospective	145	MFC	67 × 10^-5^	At relapse	21 months	OS	2.67	1.37–5.19	0.004 <0.001
An et al. (25)	Retrospective	767	CM	2%	At diagnosis	41 months	PFS OS	1.54 1.59	1.22–1.95 1.26–2.00	0.016 0.006
Vagnoni et al. (26)	Prospective	104	MFC	82 × 10^-5^	At diagnosis	35.9 months	PFS	2.63	1.51–5.92	0.004
Chakraborty et al. (17)	Retrospective	840	MFC	67 × 10^-6^	Pre-AHCT	44 months	PFS OS	2.03 2.52	1.64–2.50 1.78–3.55	<0.001 <0.001
Moor et al. (18)	Retrospective	75	MFC	10^-6^	Pre-AHCT	41 months	OS: 37.5 months vs. NR PFS: 29.5 vs. 65 months			0.043 0.017
Cowan et al. (19)	Retrospective	227	MFC	10^-4^	Pre-AHCT	Not reported	PFS OS	1.43 1.28	1.02–2.00 0.75–2.19	0.04 0.37
Sanoja-Flores et al. (11)	Retrospective	137	NGF	10^-7^	At diagnosis	Not reported	PFS	7.4	3.0–18.2	<0.0001
Galieni et al. (27)	Retrospective	168	MFC	10^-5^	At diagnosis	Not reported	PFS OS	3.18 2.79	1.54–6.59 1.31–5.96	0.002 0.008
Bertamini et al. (15)	Prospective	474	MFC	7 × 10^-4^	At diagnosis	36 months	PFS OS	2.49 2.85	1.76–3.51 1.56–5.19	<0.0001 0.0006
Jelinek 2021 (28)	Retrospective	402	MFC	2%	At diagnosis	20.5 months	PFS OS	5.8 4.2	2.8–12.4 2.1–8.6	<0.0001 <0.0001

HR higher than 1 denotes the presence of detectable gMRD to be associated with worse prognosis.

cPCs, clonal plasma cells; CI, confidence intervals; IFM, immunofluorescence microscopy; qPCR, quantative polymerase chain reaction; MFC, multiparametric flow cytometry; NGF, next-generation flow; AHCT, autologous stem cell transplantation; OS, overall survival; PFS, progression-free survival; RFS, relapse-free survival; TTP, time to progression; TTNT, time to next therapy; EFS, event-free survival; NR: not reached.

During the last two decades, many groups have tried to investigate the role of residual plasma cells in the stem cell graft. Earlier studies in the era of less intensive induction regimens the level of cPC contamination in the graft was found to contribute to myeloma relapse and progression (21, 29, 30). However, it was a greater possibility that recurrence occurred from the residual cells in the marrow weaking the role of contamination in the graft. Recently, more effective induction regimens have a potential to achieve a graft MRD (gMRD), which may serve as an additional response assessment tool. According to the recent results, higher graft purity has been found to be in concordance with deeper responses (31–33). Although gMRD is a non-invasive approach, it has not gained sufficient support for routine use. In a continuation, cPC is becoming recognized as an independent prognostic factor, and this may be an additional new tool to demonstrate the efficacy of a regimen. In view of the hurdles related to monoclonal protein assessments, high-sensitivity cellular component measurement continues to possess significant value as an end point for therapeutic efficacy.

In this review, we will present a structural framework for myeloma MRD testing in peripheral blood stem cell autografts and review the clinical integration into disease management with emphasis on future areas of research.

## Detection of Circulating Plasma Cells in Peripheral Blood Stem Cell Autografts

During recent decades, different methods have been developed and used for the detection of cPCs. Conventional cytology was first used for the identification of cPCs in blood smears at the time of diagnosis. Although they confirmed the presence of variable PC counts in a minor fraction (17%) of myeloma patients, it has not progressed beyond being a prognostic factor (34). Furthermore, limited number of nucleated cells and morphological similarities between normal and clonal cells prevent this technology to be applicable to MRD assessment (35).

The later techniques capable of greater numbers of light-chain restricted cPC quantification in the blood are as follows. Regarding a further improvement in the sensitivity and specificity of the aforementioned processes, several different conventional flow cytometry and next-generation flow cytometry (NGF) procedures and polymerase chain reaction (PCR)-based and next-generation sequencing (NGS) techniques were subsequently developed and tested in different studies (7).

Most of the gMRD-based studies utilized a well-suited conventional flow-cytometric methodology for enumeration of cPC in apheresis product (30–33, 36–38). This technique is an easy, fast, affordable, and worldwide available approach that has been extensively used to identify, characterize, and count PCs. However, the lack of standardized protocols and the highly variable sensitivity levels for detection of cPC, together with the need for fresh samples, have limited the reproducibility of results. Despite these limitations, flow cytometry has demonstrated that the presence and number of cPCs in the autograft has important clinical implications in patients with MM.

Flow cytometry studies of autograft were performed to analyze the presence of hematopoietic progenitor cells and PCs using monoclonal antibodies against CD45, CD19, CD34, CD38, and CD138 regarding the data from the study of Kopp et al. (37). Based on the results of these consecutive studies; PCs were identified with high-level expression of CD38 and CD138 (CD38^++^/CD138^+^). The number of CD34^+^ cells and CD38^++^/CD138^+^ cells/kg in the apheresis product was determined where only total PC was enumerated (36, 37). The efficacy of the single-tube seven-color flow cytometry strategy to detect rare events in apheresis samples was confirmed in a prospective study. They added anti-λ light chains, anti-κ light chains, CD28, and CD56 to a previously used panel (31). Of note, this method allows for an accurate evaluation of both normal and cPC. Patients had detectable gMRD whenever the percentage of phenotypically aberrant cPCs was equal to or greater than the limit of detection achieved in the corresponding sample. Conversely, patients had undetectable MRD when phenotypically aberrant cPCs were absent with a sensitivity of at least 10^-4^ or 10^-5^ in different studies.

Clonal B-cell populations were identified by molecular analysis of the third complementarity-determining region of the Ig heavy chain (IgH CDR3) by Ho et al. (39). Mononuclear cells from autograft underwent testing for rearrangements of IgH CDR3 using a semi-nested PCR. For patients undergoing stem cell mobilization on consecutive days, apheresis products were accepted as positive if any of the collections were positive by PCR. PCR amplification results were reported as positive in case of a clonal population of B lymphocytes consistent with cPC contamination or negative in case of a polyclonal pattern suggesting the absence of cPC contamination (39).

Recently, another emerging role of CPC has appeared in the field of precursor plasma cell disorders. As listed in **Table 2**, CPC content is associated with higher rates of transformation to MM among patients presenting with smoldering MM. These groups have identified CPC as a manifestation of high tumor burden (40–42).

**Table 2:** Studies on the impact of CPCs on progression to myeloma among patients with precursor disease.

Reference	Study design	Study population	Number of patients	Detection method	Cutoff of cPCs	Survival outcomes	HR	p value
Garces et al. (40)	Prospective	SMM, NDMM, RRMM	1,157	NGF	0.02%	PFS	1.43	0.003
Dutta et al. (41)	Prospective	MGUS, SMM	185	IFM		Intermediate-risk SMM High-risk SMM		0.00005 0.037
Oskarsson et al. (42)	Prospective	MGUS, SMM, MM	189	NGF	20 cells	MGUS: 17.8% SMM: 74% MM: 97.2%		<0.01 <0.001

HR higher than 1 denotes the presence of detectable gMRD to be associated with worse prognosis.

cPCs, clonal plasma cells; MGUS, monoclonal gammopathy of undetermined significance; SMM, smoldering multiple myeloma; NDMM, newly diagnosed multiple myeloma; RRMM, relapse/refractory multiple myeloma; IFM, immunofluorescence microscopy; NGF, next-generation flow; PFS, progression-free survival.

## Current Evidence on the Role of Graft Minimal Residual Disease Assessment in Multiple Myeloma

From the early days of autologous stem cell transplantation (AHCT), the contamination of autologous grafts has been implicated among the causes of relapse. During the era even when induction regimens were much less effective than today, contamination of apheresis products with PCs has been reported to decrease overall survival (OS) and progression-free survival (PFS) (21, 29, 30).

### Is Graft Minimal Residual Disease a Reflection of Depth of Response?

This question has not been completely answered from the gMRD perspective yet. Among all nine studies listed in **Table 3**, three of them, including ours, have analyzed the correlation between gMRD and hematological response. Wuilleme et al. (31) reported among 43 patients no correlation at a sensitivity of 10^-4^. Kopp et al. (37) reported lack of response among patients with high gMRD content. In our series presented at ASH 2021, we were able to demonstrate a correlation between CR and both gMRD (-) at level 10^-4^ [kappa coefficient (SE): -284, p = 0.03] and marrow MRD (mMRD) assessed at 10^-5^ level to correlate with CR (SE: -0.452, p = 0.001) among 411 patients (33).

**Table 3:** Studies on analysis of graft clonal plasma cell content (gMRD).

Reference	Study design	Number of patients	Detection method	Cutoff of cPCs	Follow-up	Survival outcomes	HR	95% CI	p value
Gertz et al. (21)	Prospective	33	IFM	0.2 × 10^6^/L	Not reported	RFS: 6.2 vs. 22.5 months OS			0.008 0.078
Boccadoro et al. (30)	Prospective	64	IFM	4.85 × 10^6^/kg	Not reported	EFS: 36.4 vs. 34.2 months OS: NR vs. NR			0.7
Vogel et al. (36)	Retrospective	76	MFC	4.5 × 10^-5^	Not reported	PFS: 14 vs. 26 months			0.0096
Ho et al. (39)	Retrospective	69	PCR			PFS OS			0.77 0.91
Kopp et al. (37)	Prospective	60	MFC	4.5 × 10^-5^	Not reported	PFS: 33.5 vs. 47 months OS: 53 vs. 114 months			0.15 0.012
Wuilleme et al. (31)	Prospective	53	MFC	1 × 10^-4^	15 months	PFS: 16 months vs. NR			0.008
Waszczuk-Gajda et al. (38)	Retrospective	59	MFC	2.4 × 10^-6^		Risk of progression Risk of death	1.08 1.10	1.01–1.16 1.02–1.17	0.034 0.021
Bal et al. (32)	Prospective	275	MFC	10^-5^		gMRD (-) in VRD vs. KRD groups: 57.1% vs. 81.4%			0.25
Cengiz Seval et al. (33)	Prospective	411	MFC	10^-4^		PFS OS	1.47 1.08	1.07–2.02 0.82–1.40	0.016 0.574

HR higher than 1 denotes presence of detectable gMRD to be associated with worse prognosis.

cPCs, clonal plasma cells; CI, confidence intervals; IFM, immunofluorescence microscopy; PCR, polymerase chain reaction; MFC, multiparametric flow cytometry; OS, overall survival; PFS, progression-free survival; VRD, bortezomib, lenalidomide, dexamethasone; KRD, carfilzomib, lenalidomide, dexamethasone; NR, not reached.

There are five studies in which pre-AHCT circulating cPCs have been detected and found to shorten PFS and OS. According to the results of Moor et al. (18), the real value of achieving CR relied on the MRD status, since patients in CR but without undetectable cPCs have a similar outcome to those in PR. The depth of response is strongly associated with survival, with maximum benefit for patients achieving undetectable cPCs and CR (18). In addition, Chakraborty et al. (17) also found the lower incidence of ≥very good partial response (VGPR) at the time of transplant among patients with cPCs compared with those without (22% vs. 47%, respectively; p < 0.001). However, Korthals et al. (23) among only 21 patients could not demonstrate a correlation between remission rates and level of cPCs in the blood neither before or 3 months after AHCT.

### Role of Graft Minimal Residual Disease as a Predictor of Overall Survival and Progression-Free Survival

Furthermore, few studies have attempted to quantify the level of CPC contamination in the graft. In 1995, Dreyfus et al. (29) could not show the effect of the presence of tumor cells in the apheresis product on response to AHCT; however, an early analysis of PFS provides four of eight patients with contamination who have relapsed compared with only two of 13 with undetectable gMRD. This was the first study that evaluated the impact of gMRD on outcome of AHCT (29). After Gertz et al. (21) confirmed this trend and demonstrated that the myeloma patients with higher CPC (≥0.2 × 10^6^ cells/l) in the autograft had a significantly shorter median PFS than those with low or no CPC [6.2 months vs. 22.5 months (p = 0.008, log-rank; p = 0.04, Gehan-Wilcoxon)]. In addition, they showed a trend toward improved OS in patients with detectable gMRD (p = 0.078) (21).

An earlier study during the period when induction regimens were mostly VAD (vincristine, Adriamycin, and dexamethasone) by Boccadoro et al. (30) had investigated the impact of gMRD on patient outcome, and they suggested that current *in vitro* purging techniques are unlikely to prevent myeloma recurrence; the goal remains to be *in vivo* tumor eradication. Vogel et al. (36) also reported the influence of high cPC contamination (>4.5 × 10^5^ PC/kg) in the autologous grafts on response rates and PFS of myeloma patients undergoing AHCT. The same group also found an increased risk of early disease progression among those having high graft contamination with a median PFS of 14 months (p = 0.0096). Indeed, they showed a correlation between bone marrow infiltration of cPC counts prior to AHCT and in the mobilized graft (36). In 2009, the follow-up data on the same cohort demonstrated an impact of graft contamination on OS as well (low PC: 114 months vs. high-PC: 53 months; p = 0.0012). However, PFS, although shorter, did not reach a level of statistical significance (47 months vs. 33.5 months) (37). However, the correlation between gMRD and post-AHCT survival was not confirmed by Ho et al. (39), which may be due to the methodology used being clonal IgH CDR3 gene arrangement.

The Polish MM Group was also among those interested in graft contamination reporting cPCs above 2.96 × 10^6^/kg to be correlated with shorter PFS and OS (38). In landmark analyses, a time-dependent relation was detected between cPC number and risk of death or progression. Among the patients who survived over a year after AHCT, the risk of death increased by 13% per 1 × 10^6^ of cPC number (p < 0.01), while this negative effect was not seen after the second year following AHCT (HR: 0.89; p = 0.7). In terms of PFS, the risk of progression 1 and 2 years after AHCT was not found statistically significant; however, after 3 years, positive effect of higher cPC number was observed (HR: 0.01; p < 0.054) (38). The outcome in this study was much worse in comparison to that of Vogel et al. (36); despite the differences between these studies, it can be concluded that there are myeloma patients whose further survival depends on the gMRD.

### Is Graft Minimal Residual Disease an Independent Prognostic Factor for Progression-Free Survival?

As seen in **Table 3**, gMRD has been analyzed in nine studies among which only two studies included gMRD in the multivariate analysis. In the study by Waszczuk-Gajda et al. (38), in the logistic regression analysis, presence of lambda light chain, renal failure, higher number of cPCs in bone marrow, beta-2-microglobulin, high cytogenetic risk, time period between mobilization and AHCT, hemoglobin level, and good performance status were included. They observed gMRD to correlate with mMRD (r = 0.25; p < 0.06) and more frequent among women (38). Based on our results presented at ASH 2021, among age, international staging system (ISS), cytogenetic risk, and post-induction response as significant predictors of PFS, gMRD is associated with PFS independent of age (33).

### Role of Graft Minimal Residual Disease Following Contemporary Induction Regimens

It is important to note that little is known about the efficacy of novel combinations on efficiently reducing cPC contamination within the mobilized stem cell grafts. In our single-center experience based on 102 patients for whom gMRD and mMRD assessments were possible between 2006 and 2020, we have shown that gMRD and mMRD were strongly correlated (SE: 0.638, p < 0.001), having a significant impact on post-AHCT PFS but not OS (33). When we evaluated the role of induction regimens, gMRD was detected among Daratumumab-VRD (bortezomib, lenalidomide, and dexamethasone) patients (88.9%) more frequently than those on triplet regimens (40.5%) at a sensitivity level 10^-5^ by NGF (33). gMRD after induction therapy with current frontline induction protocols [VTD (bortezomib, thalidomide, dexamethasone), VCD (bortezomib, cyclophosphamide, dexamethasone), or KRD (carfilzomib, lenalidomide, and dexamethasone)] was evaluated in another single-center prospective study (31). In this study, high response rates prior to AHCT [objective response rate (ORR): 95%] were found to be associated with undetectable gMRD measured by NGF reaching 100% median levels of normal PC and 0% median levels of cPC within apheresis products. The median PFS of patients with detectable gMRD was 16 months vs. not reached for those with undetectable gMRD (p = 0.008) (31). Nevertheless; PFS data were suggesting a potentially deleterious role for graft contamination; this has to be interpreted cautiously, as all variables were not assessed in a multivariate analysis to determine their effects on PFS.

Thus, gMRD may reflect the efficacy of induction as an additional response assessment tool. Taking into account the fact that peripheral stem cell grafts are less heterogeneous and are devoid of sampling errors adds to the ease of sampling. According to the recent results of Bal et al. (32), higher purity of graft in concordance with deeper responses was obtained with KRD rather than VRD. The clinical impact of undetectable gMRD for the patients who underwent AHCT in this study remains unknown due to insufficient follow-up from time of transplant but warrants prospective monitoring.

Considering the aforementioned studies, contamination of the graft by PCs measurable by flow cytometry might be thought to reflect residual *in vivo* tumor mass prior to AHCT but still not a part of current clinical diagnostic and treatment response criteria, which still rely on conventional biochemical, cytomorphological, immunophenotyping, molecular, and imaging criteria. An explanation for contradictory results regarding the impact of gMRD on all survival parameters is that additional important variables among sufficient number of patients have not been analyzed in a prospective manner.

To our knowledge, there is no published study that evaluates the frequency and impact of gMRD following quadruplet induction regimens. Our unpublished experience shows that all 10 Daratumumab-VRD-treated patients have reached 90% (9/10) gMRD negativity after 4 cycles. However, based on the high frequency of mMRD negativity, the frequency of gMRD negativity is expected to increase as well but remains to be confirmed (33).

## Conclusion

In recent years, several studies demonstrated MRD as a strong and reliable prognostic factor for disease progression and survival of myeloma patients. Efforts are ongoing to validate MRD assessment as a surrogate endpoint in order to accelerate the interpretation of clinical trial results. From a clinical point of view, the detection of cPC provides useful and relevant information independent of biochemical response, as plasma half-life of monoclonal proteins prevents real-time disease activity measurement. Furthermore, the size of the tumor burden does not always correlate with M protein levels. Deep-level disease quantification allows patients with high levels of cPC within graft or marrow to be offered additional cycles of induction treatment, thereby not only reducing graft contamination but also their overall tumor burden prior to AHCT. In addition, Diamond et al. (43) have recently reported clonal hematopoiesis present at myeloma diagnosis to contaminate stem cell graft and after bypass of high-dose melphalan to evolve into secondary malignancies under immunocompromised conditions. This report provides additional evidence toward the emerging importance of graft immune genotyping.

At this point, disappearance of cPC from graft, which in our experience correlates with undetectable post-induction mMRD, appears to be a powerful early predictor of PFS. Furthermore, the non-invasive nature is an additional advantage over mMRD analysis. It is important to note that if undetectable MRD is an established surrogate of prolonged survival, it may serve as an early biomarker carrying a strong predictive value.

## Author Contributions

All authors listed have made substantial, direct, and intellectual contribution to the work and approved it for publication.

## Conflict of Interest

The authors declare that the research was conducted in the absence of any commercial or financial relationships that could be construed as a potential conflict of interest.

## Publisher’s Note

All claims expressed in this article are solely those of the authors and do not necessarily represent those of their affiliated organizations, or those of the publisher, the editors and the reviewers. Any product that may be evaluated in this article, or claim that may be made by its manufacturer, is not guaranteed or endorsed by the publisher.
